# Commentary: Effect of injecting adipose stem cells combined with platelet-rich fibrin releasate at Shenshu acupoint (BL23) on acute kidney injury in rabbits

**DOI:** 10.3389/fphar.2025.1743787

**Published:** 2026-01-21

**Authors:** Xun Zheng, Ruoru Yu, Cheng Feng, Lei Wu

**Affiliations:** 1 The Third School of Clinical Medicine (School of Rehabilitation Medicine), Zhejiang Chinese Medical University, Hangzhou, China; 2 The Fourth School of Clinical Medicine, Zhejiang Chinese Medical University, Hangzhou, China; 3 Department of Acupuncture, The Third Affiliated Hospital of Zhejiang Chinese Medical University, Hangzhou, China

**Keywords:** acute kidney injury (AKI), adipose stem cells (ASCs), commentary, platelet-rich fibrin releasates (PRFrs), Shenshu acupoint

## 1 Introduction

We carefully read the article by Chuang et al. in *Frontiers in Pharmacology*. investigating the injection of adipose-derived stem cells (ADSCs) combined with platelet-rich fibrin releasate (PRFr) at the Shenshu acupoint (BL23) for treating acute kidney injury (AKI) (Chuang et al., 2025). AKI is a complex condition that impacts 10%–15% of hospitalized patients and over 50% of those in the intensive care unit (ICU) (Hoste et al., 2018). There is growing evidence that AKI is linked to a significant risk of severe short-term and long-term complications, impacts organs beyond the kidney, and leads to higher healthcare expenses (Ostermann et al., 2025). This study innovatively combines regenerative medicine materials with traditional Chinese medicine (TCM) Shenshu acupoint (BL23) injection therapy, and has confirmed a significant synergistic effect between adipose-derived stem cells (ADSCs) and platelet-rich fibrin releasate (PRFr). It break through the limitations of traditional single therapies and provide a novel therapeutic paradigm integrating traditional wisdom and modern technology for the future treatment of kidney diseases. We appreciate the innovative study by Chuang et al., which offers a novel perspective on integrating traditional Chinese medicine (TCM) with Western medicine for the treatment of kidney diseases.However, several key aspects warrant further discussion.

### 1.1 Acupoint specificity requires further validation

Although the study selected Shenshu—a classic acupoint for kidney disorders—it did not include a non-acupoint injection control. As a result, attributing the observed therapeutic effects specifically to acupoint action, rather than local diffusion alone, remains a key limitation. Without such controls, it is difficult to establish conclusively the indispensable role of the Shenshu acupoint in this therapy. Moreover, previous studies suggest that the effects of acupoint injection may be linked to specific anatomical locations and neural distributions (Zhang et al., 2014). The inclusion of non-meridian, non-acupoint control groups would help clarify the unique contribution of TCM meridian theory to stem cell therapy and exclude effects attributable solely to localized diffusion.

### 1.2 Differentiation efficiency, stability and viability of ADSCs remains unclear

Although the study confirmed migration of some ADSCs to the kidney following acupoint injection, it did not assess their differentiation efficiency, stability and viability within the target tissue. Differentiation holds profound significance in the research field of stem cells (Heng and Cao, 2004). Since acupoint injection is essentially intramuscular, comparative data on its advantages or limitations for stem cell differentiation relative to other administration routes (e.g., intravenous or direct intra-tissue injection) remain limited. Therefore, the potential benefits of acupoint injection require further validation through studies incorporating multiple administration route controls.

Since the stability and activity of adipose-derived stem cells (ADSCs) largely limit their application as therapeutic agents, supplementing post-injection stability studies would further consolidate their application value in this research. So, to evaluate the longer-term survival of ADSCs within the kidney tissue (e.g., via *in vivo* imaging beyond 1 week), their proliferation/differentiation status (via specific marker staining), and the duration of their paracrine effects are critical for assessing the therapy’s long-term efficacy and determining optimal treatment intervals.

### 1.3 The evaluation of renal injury is not sufficiently accurate and comprehensive

The evaluation of renal injury is not sufficiently accurate and comprehensive. The renal tubular brush border is composed of dense microvilli rich in glycoproteins, while hematoxylin and eosin (H&E) staining mainly visualizes proteins (via eosin) and nucleic acids (via hematoxylin), with weak binding ability to glycoproteins and low contrast. Therefore, when investigating the specific mechanism of adipose-derived stem cells (ADSCs) combined with platelet-rich fibrin releasate (PRFr) in synergistically repairing renal tubules, relying solely on H&E staining may miss key pathological information. In contrast, periodic acid-Schiff (PAS) staining, based on its principle of specifically displaying polysaccharides and glycoproteins, (Zhou et al., 2012) can more intuitively assess the degree of brush border loss or injury; it can provide high-contrast specific identification of granular or vacuolar changes caused by intracellular glycogen accumulation or abnormal metabolism of specific glycoproteins.

In addition, studies related to renal function should be more comprehensive. Adding the detection of urine albumin/creatinine ratio can more accurately reflect the occurrence of proteinuria and clarify whether the use of ADSCs alone or in combination with PRFr can reverse such renal function decline. Furthermore, detecting the levels of pro-inflammatory cytokines such as tumor necrosis factor-α (TNF-α) and interleukin-6 (IL-6) can quantitatively evaluate the anti-inflammatory effect of the treatment, providing more effective evidence for the potential paracrine immunomodulatory mechanism of ADSCs. Thus, it can more precisely reveal the physiological and pathological pathways (such as anti-inflammation, protection of the filtration barrier, and tubular repair) through which ADSCs and PRFr synergistically exert renal protective effects, making the conclusion on efficacy more mechanistically in-depth and convincing.

### 1.4 Mechanisms of action demand deeper investigation

The limited depth of mechanistic investigation constrains understanding of the therapeutic principles. The study observed synergistic effects between ADSCs and PRFr but provided limited exploration of the underlying molecular mechanisms. Several key questions remain: Did ADSCs directly differentiate into renal parenchymal cells, such as renal tubular epithelial cells, contributing to structural repair? Or did they act primarily through paracrine release of trophic factors, exosomes, etc., to modulate the local microenvironment and promote endogenous repair? Regarding the role of PRFr, does it mainly support ADSC viability, or does it act directly on damaged kidney cells? How do the multiple growth factors in PRFr interact with ADSCs? Studies have shown that platelet-rich plasma (PRP) can enhance the secretion of therapeutically active exosomes by mesenchymal stem cells (MSCs) (Ji et al., 2021), but whether such interactions occur between PRFr and ADSCs is still unclear. Additionally, further investigation is needed into key signaling pathways—such as regenerative or anti-fibrotic pathways like PI3K/Akt, Wnt/β-catenin, or ERK, and whether key inflammatory or apoptotic pathways are inhibited. Clarifying whether ADSCs contribute through differentiation or secretory mechanisms is essential for optimizing therapeutic strategies.

### 1.5 Dose-response relationship and optimization are lacking

In clinical stem cell therapy, the quantity of stem cells transfused and the highest tolerable dose for human transplantation are important considerations (Kabat et al., 2020). Determining minimal effective doses (MEDs) for various delivery methods is expected to reduce the long-term expenses of clinical trials involving many participants by concentrating on the most effective doses. So, the absence of dose optimization studies affects the reproducibility of the treatment protocol. The study used a fixed dose of ADSCs (2 × 10^6 cells) and a fixed volume of PRFr (0.5 mL), without systematic dose-escalation experiments to explore dose-response relationships or determine the optimal therapeutic dose. Furthermore, different injury severities may require tailored dosing regimens, an issue that should be addressed in future studies.

### 1.6 Long-term safety assessment is insufficient

The study duration limits the assessment of long-term safety. Although the 7-week observation period demonstrated short-term efficacy, it is insufficient for evaluating potential risks associated with stem cell therapy. Studies indicate that stem cell therapies may pose risks such as aberrant proliferation and tumorigenicity (Barkholt et al., 2013). Given that PRFr is rich in growth factors that could promote excessive cell proliferation, the lack of long-term monitoring data for fibrosis markers (e.g., α-SMA, collagen deposition) and oncogenic risk represents a significant limitation.

## 2 Discussion

**FIGURE 1:**
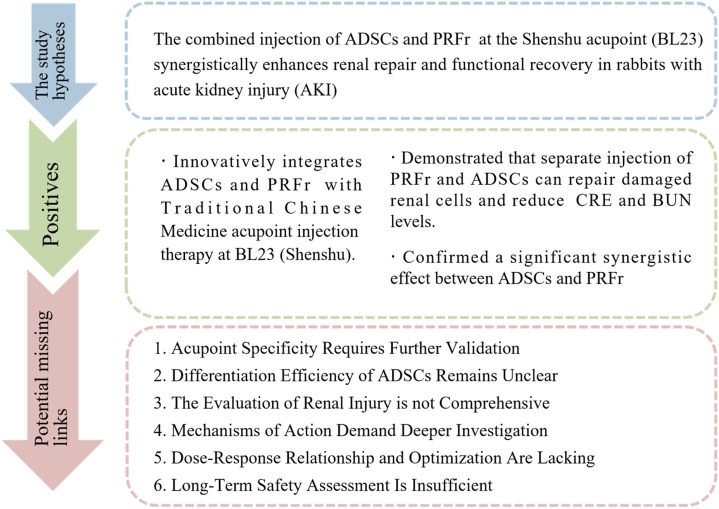
The schematic flow diagram of the commentary.

Despite these limitations (Figure 1), this study provides valuable foundational data for renal regenerative medicine. Future research should focus on validating acupoint specificity, comparing different administration routes, optimizing dosing, conducting in-depth mechanistic studies, and extending observation periods to further clarify the safety, efficacy, and mechanisms of this therapeutic approach.
